# MiR21 sensitized B-lymphoma cells to ABT-199 via ICOS/ICOSL-mediated interaction of Treg cells with endothelial cells

**DOI:** 10.1186/s13046-017-0551-z

**Published:** 2017-06-21

**Authors:** Zhong Zheng, Peng-Peng Xu, Li Wang, Hui-Jin Zhao, Xiang-Qin Weng, Hui-Juan Zhong, Bin Qu, Jie Xiong, Yan Zhao, Xue-Feng Wang, Anne Janin, Wei-Li Zhao

**Affiliations:** 1 State Key Laboratory of Medical Genomics, Shanghai Institute of Hematology, Shanghai Rui Jin Hospital, Shanghai Jiao Tong University School of Medicine, 197 Rui Jin Er Road, Shanghai, 200025 China; 2 Pôle de Recherches Sino-Français en Science du Vivant et Génomique, Laboratory of Molecular Pathology, Shanghai, China; 3 Department of Laboratory Medicine, Shanghai Rui Jin Hospital, Shanghai Jiao Tong University School of Medicine, Shanghai, China; 4 U1165 Inserm/Université Paris 7, Hôpital Saint Louis, Paris, France

**Keywords:** MicroRNA21, B-cell lymphoma, ABT-199, Tumor microenvironment, Regulatory T cells, Endothelial cells

## Abstract

**Background:**

MicroRNAs (miRs) are involved in tumor progression by regulating tumor cells and tumor microenvironment. MiR21 is overexpressed in diffuse large B-cell lymphoma (DLBCL) and its biological impact on tumor microenvironment remains unclear.

**Methods:**

MiR21 was assessed by quantitative RT-PCR in patients with newly diagnosed DLBCL. The mechanism of action of miR21 on lymphoma progression and tumor angiogenesis was examined in vitro in B-lymphoma cell lines and in vivo in a murine xenograft model.

**Results:**

Serum miR21 was significantly elevated in patients and associated with advanced disease stage, International Prognostic Index indicating intermediate-high and high-risk, and increased tumor angiogenesis. When co-cultured with immune cells and endothelial cells, miR21-overexpressing B-lymphoma cells were resistant to chemotherapeutic agents, but sensitive to Bcl-2 inhibitor ABT-199, irrespective of Bcl-2 expression on lymphoma cells. In both co-culture systems of Bcl-2^positive^ and Bcl-2^negative^ B-lymphoma cells, miR21 induced inducible co-stimulator (ICOS) expression on regulatory T (Treg) cells. Through crosstalking with Treg cells by ICOS ligand (ICOSL), endothelial cells were activated, resulting in stimulation of Bcl-2 expression and vessel formation. ABT-199 directly targeted Bcl-2 on endothelial cells, induced endothelial cell apoptosis and inhibited tumor angiogenesis. In a murine xenograft model established with subcutaneous injection of B-lymphoma cells, ABT-199 particularly retarded the growth of miR21-overexpressing tumors, consistent with the induction of endothelial cell apoptosis and inhibition of tumor angiogenesis.

**Conclusions:**

As a serum oncogenic biomarker of B-cell lymphoma, miR21 indicated B-lymphoma cell sensitivity to ABT-199 via ICOS/ICOSL-mediated interaction of Treg cells with endothelial cells.

**Electronic supplementary material:**

The online version of this article (doi:10.1186/s13046-017-0551-z) contains supplementary material, which is available to authorized users.

## Background

Diffuse large B-cell lymphoma (DLBCL) represents the most common neoplastic disorder of B-lymphocytes. Although addition of anti-CD20 antibody Rituximab to conventional chemotherapy has significantly improved the clinical outcome of the patients, due to disease heterogeneity, relapse and refractory to immunochemotherapy remains of great concern [1]. As a newly developed bio-therapeutic agent, ABT-199 is a specific Bcl-2 inhibitor targeting the BH3-binding domains of Bcl-2 and has demonstrated encouraging preclinical results in treating B-cell lymphoma [2, 3]. However, the underlying mechanism of ABT-199 in treating DLBCL warrants further investigation.

Besides genetic abnormalities of malignant cells themselves, the aberrant status of tumor microenvironment plays a pivotal role on disease progression [4]. As the main components of microenvironment, tumor vessels prevent the attack of chemotherapy on tumor cells [5]. Moreover, tumor angiogenesis mediated by immune cells is involved in the development of drug resistance in lymphoma [6, 7]. Direct interaction of immunosuppressive regulatory T (Treg) cells with vascular endothelial cells contributes to the regulation of anti-lymphoma responses [8, 9]. Inducible co-stimulator (ICOS) and its ligand ICOSL function as an important crosstalk between immune cells and endothelial cells [10]. Since it has recently been reported that the cytotoxic effect of ABT-199 on tumor cells rely on survival signals from tumor microenvironment [11], microenvironment-related biomarkers may become potential predictors of ABT-199 efficacy on B-cell lymphoma.

MicroRNAs (miRs), a class of 19- to 23-nucleotide non-coding RNA molecules, regulate gene expression by targeting mRNA at the 3’-untranslated region [12]. In addition to their oncogenic action on tumor cells mediated by oncogenes and/or tumor suppressor genes, miRs are emerging as key regulators of tumor microenvironment [13, 14]. Among them, miR21 can target Bcl-2 expression by binding to Bcl-2 3’-untranslated region (610–617 bp) and stably expressed in peripheral blood and closely related to disease outcome in B-cell lymphoma as previously reported [15–17]. In the present study, we assessed serum miR21 expression in a large cohort of DLBCL patients and revealed the biological function of miR21 on tumor microenvironment both in vitro and in vivo. Although potentially oncogenic, miR21 enhanced the sensitivity of B-lymphoma cells to ABT-199, through an alternative mechanism involving tumor angiogenesis.

## Methods

### Patients

A total of 203 patients with newly diagnosed DLBCL were enrolled in this study. The main clinical characteristics of the patients were listed in Table 1. Paraffin tumor samples of 50 DLBCL patients were used for immunohistochemistry study. One hundred healthy volunteers were referred as normal control. The study was approved by the Shanghai Rui Jin Hospital Review Board with informed consent obtained from all subjects in accordance with the Declaration of Helsinki.

**Table 1:** Clinical characteristics of patients with DLBCL (*n* = 203)

	High miR21	Low miR21	*P*-value
Age			
> 60 years	35	39	0.523
≤ 60 years	67	62	
Sex			
Female	39	41	0.731
Male	63	60	
Ann Arbor stage			
I to II	50	65	0.027
III to IV	52	36	
Extranodal involvement			
No	18	17	
Yes	84	84	0.882
Serum LDH level			
Normal	53	68	0.071
Abnormal	49	33	
IPI score			
Low and intermediate-low risk	59	75	0.013
Intermediate-high and high risk	43	26	

*LDH* lactate dehydrogenase, *IPI* International Prognostic Index

### Cells and reagents

Human B-lymphoma cell lines SU-DHL-4, SU-DHL-8, human umbilical vein endothelial cell (HUVEC), and murine B-lymphoma cell line A20 were obtained from American Type Culture Collection (Manassas, VA, USA). Cells were cultured in humidified atmosphere of 95% air and 5% CO_2_ at 37 °C. ABT-199 was purchased from Selleck-Biotool (Houston, TX, USA). Anti-Human ICOS functional grade purified antibody was from Affymetrics Ebioscience (San Diego, CA, USA).

### Serum and tissue miR21 detection

Total serum miRNA was extracted using miRNeasy Serum/Plasma Kit (Qiagen, Valencia, CA, USA). MiR21 was measured by real-time quantitative RT-PCR using miScript reverse transcription kit, hsa-miR21 primer and miScript SYBR Green PCR kit (Qiagen). MiR39 was used as endogenous control and DB cells for calibration. Total tissue miRNA was extracted using Trizol agent (Invitrogen, Carlsbad, CA, USA). RNU6 was used as endogenous control and DB cells for calibration. The reactions were analyzed on 7500HT Fast Real-time PCR system (Applied Biosystem, Carlsbad, CA, USA). A relative quantification was calculated using the ^2-ΔΔ^CT method.

### Cell proliferation assay

Cell proliferation assay was performed as previously described [18].

### In vitro co-culture system

Transwell cell culture chambers (1 μM, Millipore Corporation, Billerica, MA, USA) were used for co-culture assay. In the co-culture system, lymphoma cells were plated on the upper chamber, with immune cells and HUVEC monolayer on the lower chamber, allowing direct contact of HUVEC with immune cells. Immune cells were mononuclear cells isolated from peripheral blood of healthy volunteers using Ficoll by density gradient centrifugation.

### Cell transfection

SU-DHL-4 and SU-DHL-8 cells were transfected with miR21 mimics (Riobio, Guangzhou, China) or negative control (Riobio) using lipofectamine 2000 (Invitrogen) following the manufacturer’s instruction. For the knock-down assay, SU-DHL-4, SU-DHL-8 cells, and HUVEC were transfected with Bcl-2 siRNA or control siRNA (Origene, Rockville, MD, USA) using lipofectamine 2000.

#### Luciferase report assay

HEK-293 T cells were transfected with luciferase reporter and miR21 mimics, using Lipofectamine 2000 (Invitrogen) according to the manufacturer’s instructions. Protein was collected 24 h after transfection, using the Passive Lysis Buffer (30 μL per well) provided as part of the Dual-Luciferase Reporter Assay System kit (Promega). Firefly and Renilla luciferase activities were examined by the Dual-Luciferase Reporter Assay System and detected by a Centro XS3 LB960 Luminometer (Berthold).

### Lentivirus packaging and transduction

To overexpress miR21 in A20 cells, purified plasmids pGMLV-miR21 or control vector were transfected into HEK-293 T cells with package vectors using lipofectamine 2000. The supernatant of HEK-293 T cell culture was then condensed to a viral concentration of approximately 3 × 10^8^ transducing units/ml. The lentiviral particles were incubated with A20 cells for 8 h. The stably transduced cells were selected by green fluorescence protein.

### Flow cytometry

SU-DHL-4 cells were sorted by EasySep™ Human CD20+ Cell Isolation Kit, Treg cells by EasySep™ Human CD4 + CD127lowCD25+ Regulatory T Cell Isolation Kit (STEMCELL, Vancouver, BC, Canada), and HUVEC by CD31 microbeads kit (Miltenyi Biotec. Shanghai, China). Purity of the sorted populations was greater than 98%. ICOS expression on Treg cells was assessed using anti-ICOS antibody (Abcam, Cambridge, UK) as the primary antibody and goat anti-mouse IgG H&L (Abcam) as the secondary antibody. ICOSL expression on HUVEC was assessed using anti-ICOSL antibody (Abcam) as the primary antibody and donkey anti-rabbit IgG H&L (Abcam) as the secondary antibody. The median fluorescent intensity (MFI) was measured by flow cytometry. Cell cycle and apoptosis analysis were conducted as previously described [19].

### Western blot

Cells were collected and lysed in 200 μL lysis buffer (Sigma Aldrich, Shanghai, China). Protein lysates (20 μg) were electrophoresed on 10% sodium dodecylsulfate polyacrylamide gels and transferred to nitrocellulose membranes. Membranes were blocked with 5% non-fat dried milk and incubated overnight at 4 °C with appropriate primary antibody, followed by horseradish peroxidase-linked secondary antibody. The immunocomplexes were visualized using chemiluminescence phototope-horseradish peroxidase Kits. Antibodies against Bcl-2, PDCD4, STAT3, p-STAT3 were from Cell Signaling Technology (Danvers, MA, USA). β-actin was used to ensure equivalent loading of cell protein.

### TUNEL assay

In situ cell apoptosis was determined with detection of fragmented DNA, using in situ cell death detection kit (Roche, Shanghai, China) according to the manufacturer’s instructions.

### Tube formation assay

96-well plates were coated with a thin layer of the Matrigel (BD Biosciences, CA, USA) and left to polymerize at 37 °C for 0.5 h. HUVEC (2 × 10^4^ cells/well) were starved overnight before being resuspended in 100 μL endothelial cell medium and added to the polymerized Matrigel. After 8 h incubation, tube formation ability was assessed by calculating total tube length in three random fields using Wimasis Image Analysis Platform.

### Immunohistochemistry and immunofluorescence assay

Immunohistochemistry was performed on 5 μm-paraffin sections with an indirect immunoperoxidase method using the primary antibody against FOXP3 (1:400, Cell Signaling, Beverly, MA, USA), CD31 (1:400, Abcam), p-STAT3 (1:400, Cell Signaling) and Bcl-2 (1:400, Cell Signaling). Microvessel density was scored semi-quantitatively based on expression levels of CD31 positive cells, +/++ being some staining or rare vessel lumens, +++/++++ being some or more vessel lumens [20]. Immunofluorescence assay was performed on methanol-fixed cells or 5 μm-frozen sections using antibody against ICOS and ICOSL (Abcam). Texas red conjugated donkey anti-rabbit IgG antibody and FITC-conjugated goat anti-mouse IgG (Abcam) were used as the secondary antibody.

### Murine model

To test the in vivo efficiency of ABT-199, BALB/c mice (5–6-week-old, obtained from Shanghai Laboratory Animal Center, Shanghai, China) were injected with 1 × 10^7^ A20 cells into the right flank. Treatments started after tumor became about 0.5 cm × 0.5 cm in surface (Day 0). For oral treatment, ABT-199 (10 mg/ml) was formulated in 60% phosal 50 propylene glycol, 30% polyethyleneglycol-400 and 10% ethanol. Tumor volumes were calculated as 0.5 × a(length) × b(width) ^2^.

### Transmission electron microscopy

Ultrastructural studies focused on the morphology of tumor microvessels as previously described [19].

### Statistical analysis

Difference of miR21 expression among groups was calculated using Mann–Whitney *U* test. In vitro experimental results were expressed as mean ± S.D. of data obtained from three separate experiments and determined by *t*-test to compare variance. Statistical significance was defined as *p* < 0.05.

## Results

### Serum miR21 was elevated in B-cell lymphoma and indicated lymphoma progression

**Fig. 1:**
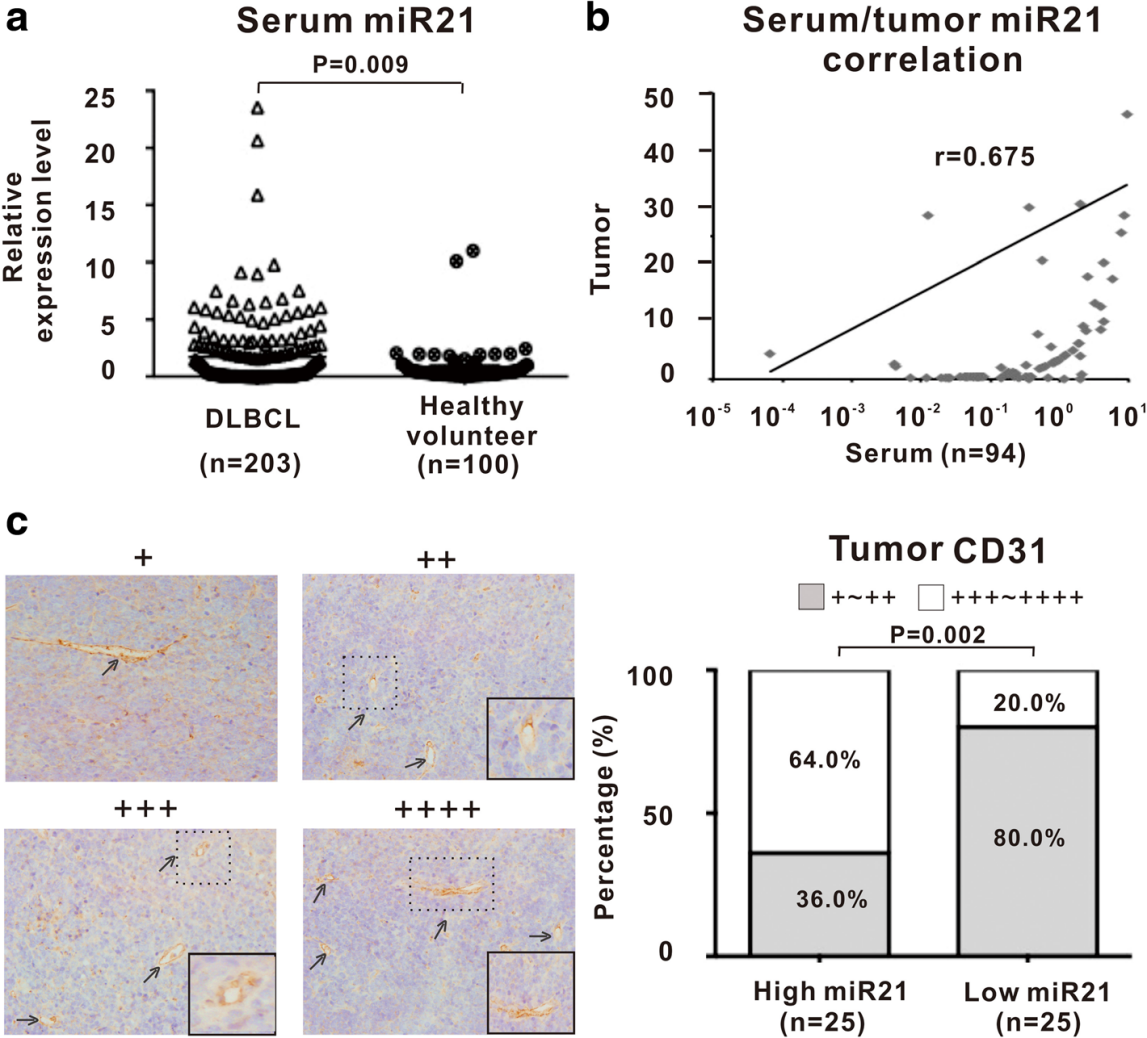
Serum miR21 was elevated in B-cell lymphoma and indicated lymphoma progression. **a** Serum miR21 was significantly higher in DLBCL patients than in health volunteers. The relative expression level of each patient was calculated based on the lowest expression value. **b** A significant correlation was observed between serum and tumor miR21 expression level. Correlation coefficient was determined by Pearson correlation coefficient analysis. **c** Patients with high serum miR21 expression displayed significantly increased tumor CD31 positivity. The patients with miR21 expression level over and equal to the median value were regarded as high miR21 expression, while those below to the median value were included into the low miR21 expression

Comparing with healthy volunteers, serum miR21 was significantly increased in patients with DLBCL (*P* = 0.009, Fig. 1a). A significant correlation between serum and tumor miR21 expression was observed by Pearson correlation coefficient analysis (r = 0.675, Fig. 1b). Elevated miR21 levels were associated with advanced Ann Arbor stage and International Prognostic Index indicating intermediate-high and high-risk (*P* = 0.027 and *P* = 0.013, Table 1). The median expression of miR21 was 0.318 in DLBCL. The patients with miR21 expression level over and equal to the median value were regarded as high miR21 expression, while those below the median value were included into low miR21 expression. As revealed by immunohistochemistry in tumor samples of DLBCL (25 each with high or low miR21 expression), CD31-positive microvessels were more frequently observed in high miR21 group than in low miR21 group (*P* = 0.002, Fig. 1c). These data suggested that serum miR21 was related to tumor progression and tumor angiogenesis in B-cell lymphoma.

### MiR21 overexpression enhanced B-lymphoma cell chemoresistance but ABT-199 sensitivity involving tumor microenvironment

**Fig. 2:**
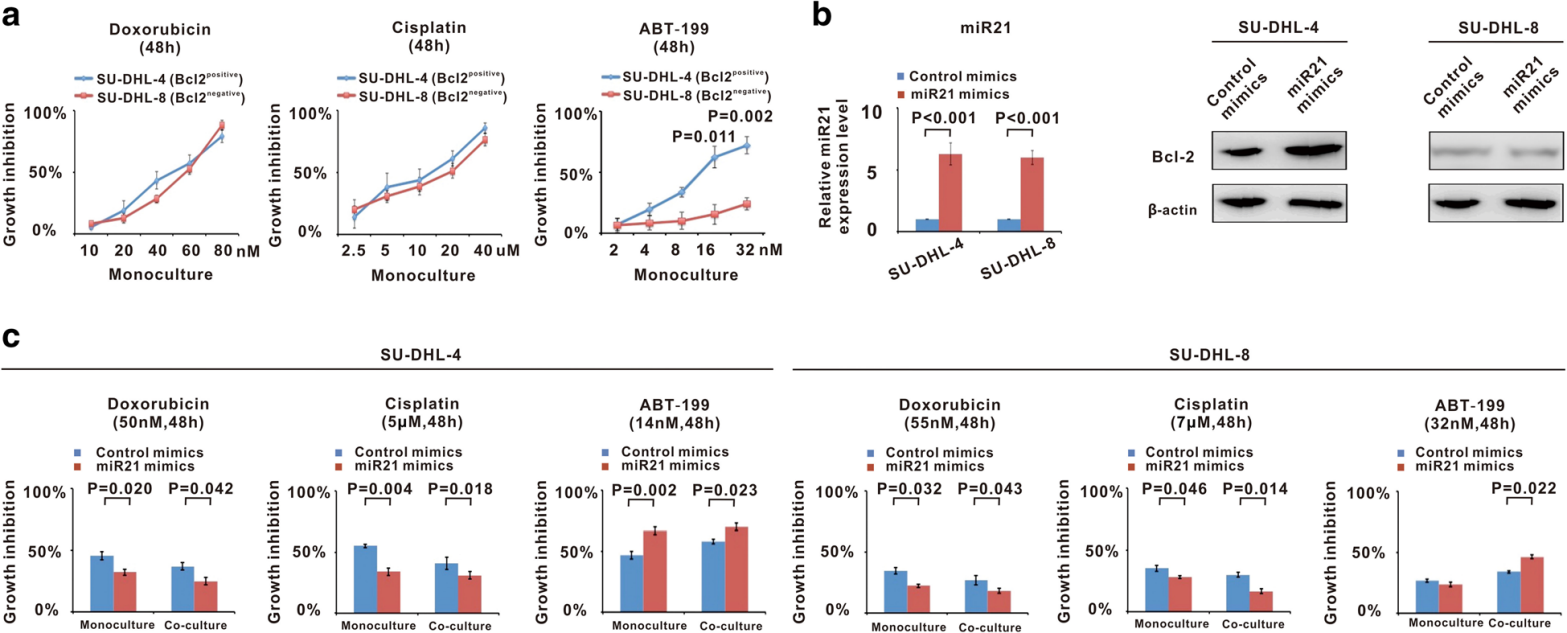
MiR21 overexpression enhanced B-lymphoma cell chemoresistance but ABT-199 sensitivity involving tumor microenvironment. **a** Cell growth was determined by MTT assay under the monoculture condition of SU-DHL-4 and SU-DHL-8 cells treated with doxorubicin, cisplatin and ABT-199. **b** Comparing with the control mimics, transfection with miR21 mimics in SU-DHL-4 and SU-DHL-8 cells resulted in significantly increased miR21 expression. MiR21 overexpression induced Bcl-2 expression on SU-DHL-4, but not on SU-DHL-8. **c** MiR21 overexpression induced lymphoma cell resistance to doxorubicin (50 nm in SU-DHL-4, 55 nm in SU-DHL-8) or cisplatin (5 μM in SU-DHL-4, 7 μM in SU-DHL-8) in the monoculture system and in the co-culture system, but sensitivity to ABT-199 (14 nm in SU-DHL-4, 32 nm in SU-DHL-8) in the co-culture system

Bcl-2^postive^ B-lymphoma cells SU-DHL-4 and Bcl-2^negative^ B-lymphoma cells SU-DHL-8 were treated with different concentrations of doxorubicin, cisplatin and ABT-199. Dose-response curves were shown in Fig. 2a. In the monoculture condition, compared with doxorubicin and cisplatin, the IC50 of ABT-199 in SU-DHL-8 was unachievable, confirming that the cytotoxic effect of ABT-199 relied on Bcl-2 expression in tumor cells. Accordingly, cell cycle arrest and cell apoptosis were observed in SU-DHL-4 cells, but not in SU-DHL-8 cells (Additional file 1: Figure S1A and B). To determine the biological function of miR21, SU-DHL-4 and SU-DHL-8 cells were transfected with miR21 mimics. Western blot showed that Bcl-2 protein level was increased in SU-DHL-4, while that of SU-DHL-8 was not inducible as previously reported [21] (Fig. 2b). While mimicking lymphoma microenvironment, cell proliferation assays were further performed in the co-culture system of lymphoma cells with immune cells and HUVEC cells. Different from the monoculture condition, miR21 overexpression resulted in lymphoma cell resistance to chemotherapeutic agents, but its sensitivity to ABT-199, is found not only in the co-culture system of Bcl-2^postive^ SU-DHL-4 cells, but also in the co-culture system of Bcl-2^negative^ SU-DHL-8 cells (Fig. 2c). Therefore, irrespective of lymphoma cell Bcl-2 status, miR21 sensitized B-lymphoma cells to ABT-199 in the presence of tumor microenvironment.

### MiR21 induced ICOS expression on Treg cells through p-STAT3 upregulation

**Fig. 3:**
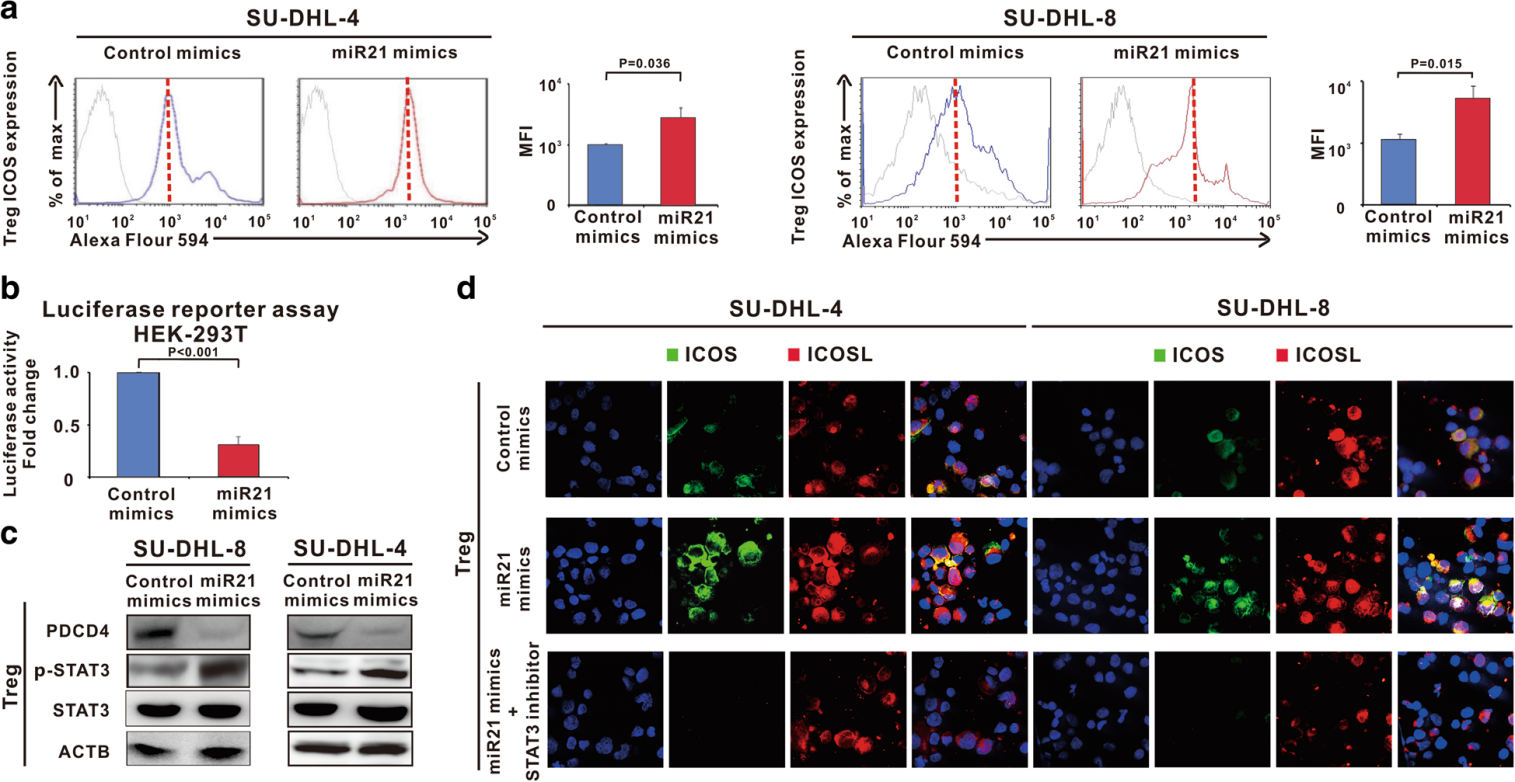
MiR21 induced ICOS expression on Treg cells through p-STAT3 upregulation. **a** Treg cells were sorted from the co-culture system for further study, ICOS expression on Treg cells was increased by miR21 overexpression in the co-culture systems of SU-DHL-4 and SU-DHL-8 cells**. b** The effect of miR21 on transcriptional activity of the PDCD4 promoter was measured by luciferase reporter assay in HEK-293 T cells transfected with control mimics or miR21 mimics. **c** Downregulation of PDCD4 and upregulation of p-STAT3 were detected in Treg cells. **d** Increased ICOS expression on Treg cells was blocked by the STAT inhibitor (Representative immunofluorescene images of ICOS (*green*)/ICOSL (*red*) with cells counterstained with DAPI (*blue*))

To clarify the underlying mechanism behind miR21-mediated sensitization of ABT-199 on B-cell lymphoma, Treg cells were sorted from the co-culture system and the effect of tumor miR21 on Treg cells was studied. Ectopic expression of miR21 of lymphoma cells significantly increased ICOS expression on Treg cells in both co-culture systems of SU-DHL-4 and SU-DHL-8 cells (*P* = 0.036 and *P* = 0.015, Fig. 3a). Previous study reported that miR21 regulates tumor progression through the miR21-PDCD4-STAT3 pathway [22]. As shown by luciferase reporter assay, miR21 repressed the transcriptional activity of the PDCD4 promoter (228–249 bp) in HEK-293 T cells (Fig. 3b). In Treg cells, PDCD4 was downregulated and phosphorylation of STAT3 was upregulated (Fig. 3c). Meanwhile, pharmacological inhibition of STAT3 by the STAT3 inhibitor abrogated the increased expression of ICOS on Treg cells induced by miR21 overexpression (Fig. 3d). Thus, miR21-mediated p-STAT3 phosphorylation was necessary for the induction of ICOS expression on Treg cells, independent of Bcl-2 expression of lymphoma cells.

### ABT-199 counteracted miR21-mediated tumor angiogenesis through ICOS/ICOSL-mediated interaction of Treg cells with endothelial cells

**Fig. 4:**
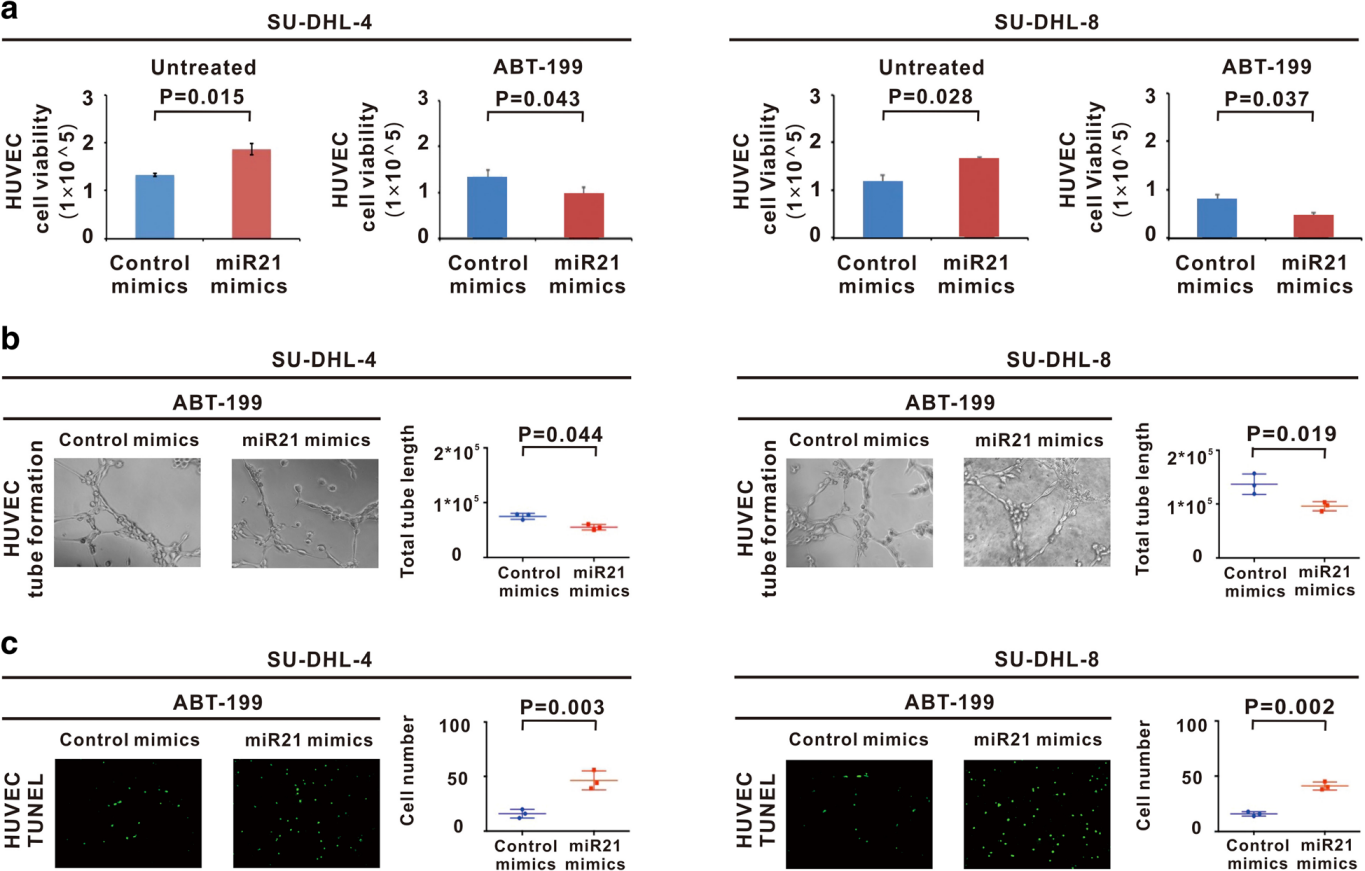
ABT-199 counteracted miR21-mediated angiogenesis. **a** HUVEC cells were sorted from the co-culture system for further study, HUVEC growth was enhanced by miR21 overexpression and reduced by ABT-199 treatment in the co-culture systems of SU-DHL-4 and SU-DHL-8 cells. **b-c** ABT-199-mediated inhibition of HUVEC growth was associated with decreased tube formation (**b**) and increased HUVEC apoptosis, as revealed by TUNEL assay (**c**)

HUVEC cells were also sorted from the co-culture system. In both co-culture systems of SU-DHL-4 and SU-DHL-8 cells, ICOSL was stably expressed on HUVEC (Additional file 2: Figure S2). MiR21 overexpression of lymphoma cells significantly enhanced HUVEC growth (*P* = 0.015 and *P* = 0.028), which was retarded upon ABT-199 treatment (*P* = 0.043 and *P* = 0.037, Fig. 4a). Accordingly, vessel formation was reduced by ABT-199 (*P* = 0.044 and *P* = 0.019, Fig. 4b), in association with increased percentage of TUNEL positive endothelial cells (*P* = 0.003 and *P* = 0.002, Fig. 4c).

**Fig. 5:**
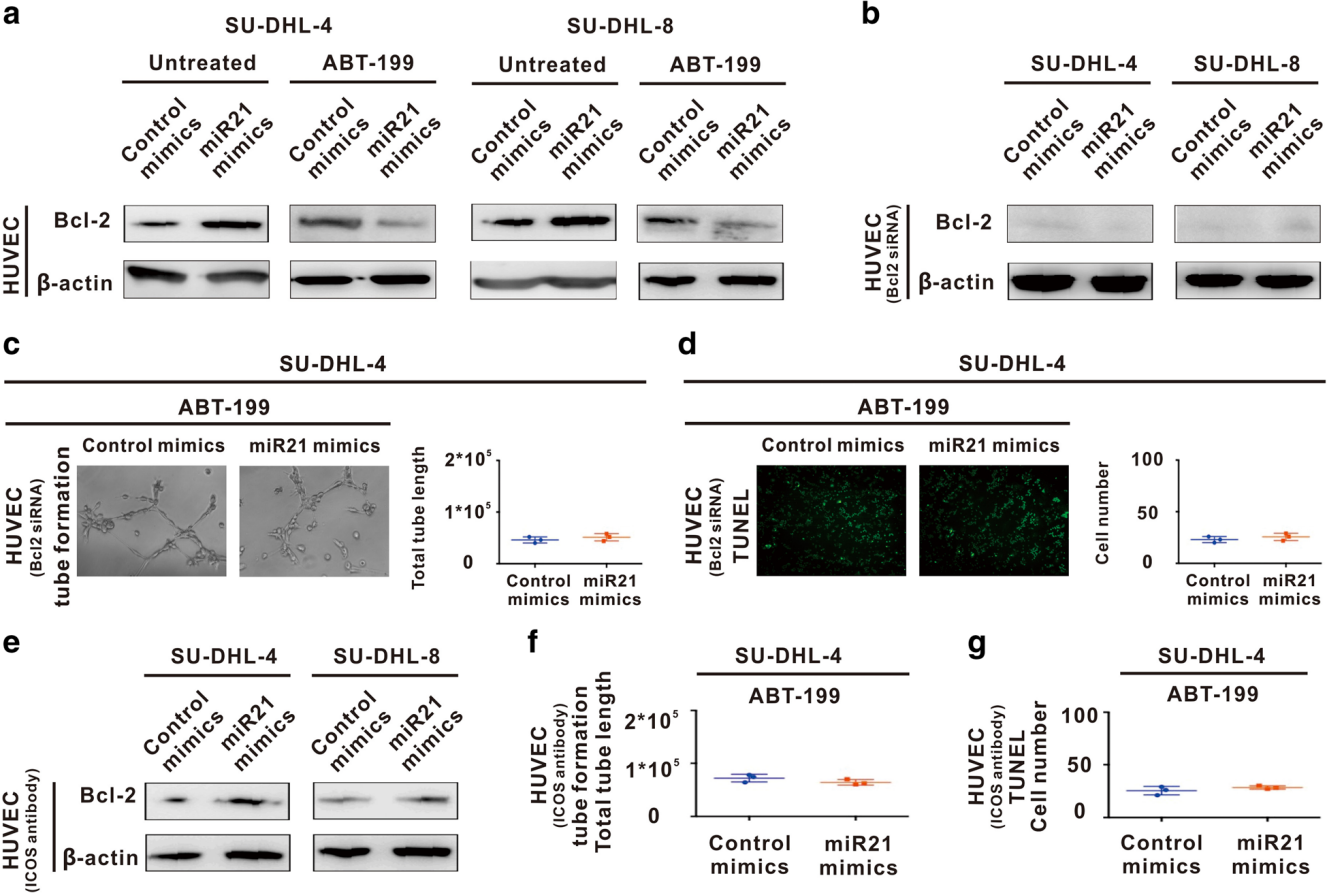
ABT-199 targeted Bcl-2 on endothelial cells and inhibited angiogenesis. **a** HUVEC cells were sorted from the co-culture system for further study, Bcl-2 expression on HUVEC was increased by miR21 overexpression and decreased by ABT-199 treatment in the co-culture system of SU-DHL-4 and SU-DHL-8 cells. **b** HUVEC were transfected with Bcl-2 siRNA. **c-d** Molecular silencing of Bcl-2 in HUVEC abrogated ABT-199-mediated reduction of tube formation (**c**) and induction of cell apoptosis (**d**). **e-g** Addition of ICOS antibody in HUVEC counteracted ABT-199-mediated reduction of Bcl-2 expression (**e**), inhibition of tube formation (**f**) and induction of cell apoptosis (**g**)

Of note, Bcl-2 expression was increased on HUVEC co-cultured with miR21-overexpressing SU-DHL-4 cells or SU-DHL-8 cells, which was downregulated upon ABT-199 treatment (Fig. 5a). Bcl-2 was further knocked down by siRNA in HUVEC (Fig. 5b). As detected by tube formation assay and TUNEL assay, the effects of ABT-199 on HUVEC was abrogated by molecular silencing of Bcl-2 (Fig. 5c and d). Meanwhile, Bcl-2 expression remained constant when ICOS antibody was added to block the interaction between Treg cells and HUVEC (Fig. 5e). Similar results were obtained on tube formation and HUVEC apoptosis (Fig. 5f and g).

Together, miR21 increased interaction of Treg cells with endothelial cells via ICOS/ICOSL axis and stimulated tumor angiogenesis, which was interrupted by ABT-199 through targeting Bcl-2 expression on endothelial cells.

### ABT-199 exhibited in vivo activity on miR21-overexpressing lymphoma

**Fig. 6:**
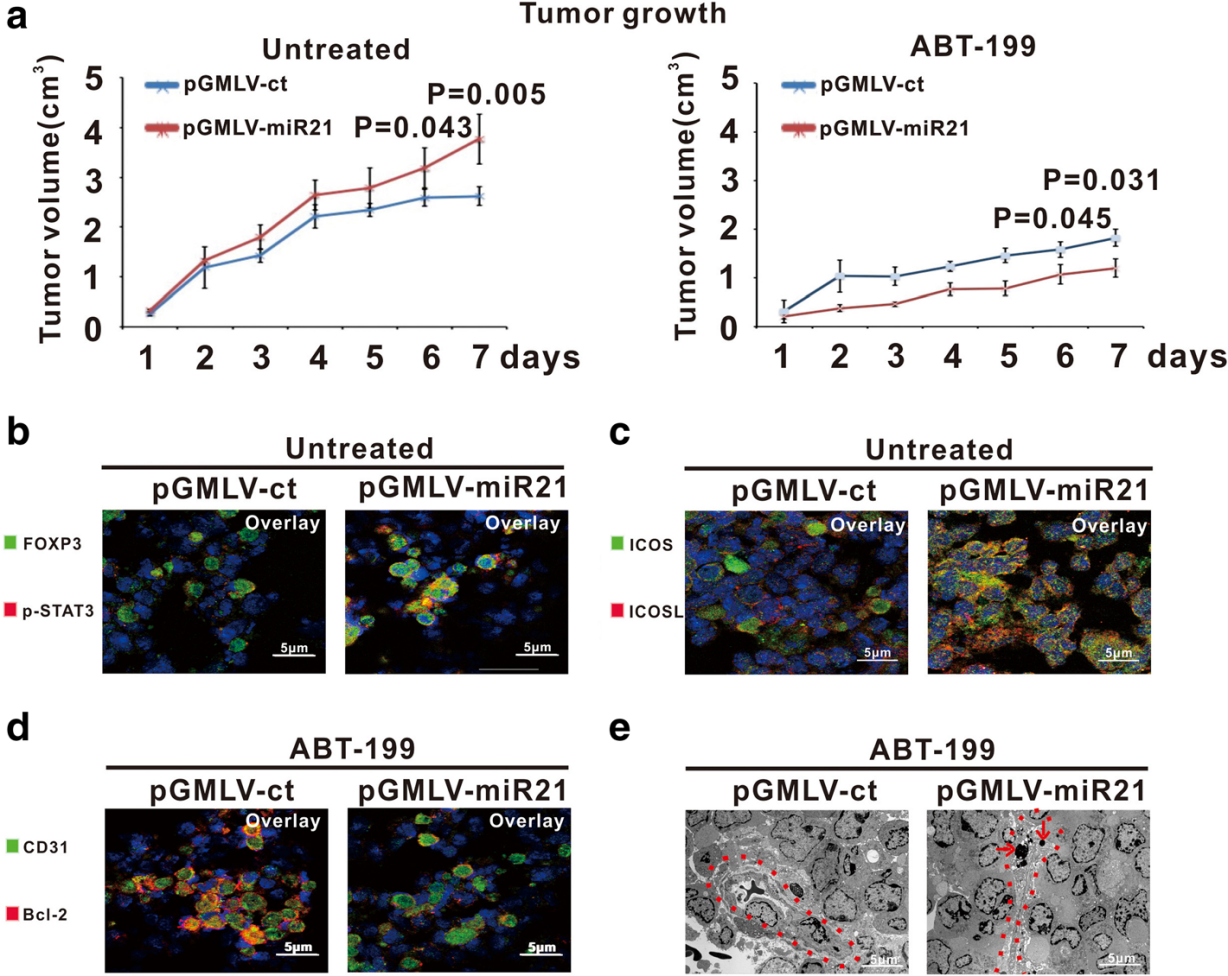
ABT-199 exhibited in vivo activity on miR21-overexpressing lymphoma. **a** MiR21-overexpressing pGMLV-miR21 tumors grew more quickly than the control pGMLV-ct tumors and were more sensitive to ABT-199 treatment. **b**-**c** p-STAT3-positive Treg cells (**b**) and ICOS/ICOSL interaction (**c**) were decreased in untreated pGMLV-miR21 tumors. **d**-**e** Bcl-2 expression (**d**) and cell apoptosis revealed by ultrastructural analysis (**e**) were increased in microvessels of ABT-199-treated pGMLV-miR21 tumors. Microvessels were indicated by *red broken lines* and apoptotic bodies by *red arrow*

Murine xenograft model was established with subcutaneous injection of A20 cells either stably transfected with pGMLV-miR21 or control vector pGMLV-ct. The tumor size of pGMLV-miR21 group was significantly larger than that of pGMLV-ct group (*P* = 0.043 at Day 6 and *P* = 0.005 at Day 7, Fig. 6a). ABT-199 treatment particularly exhibited anti-tumor activity on pGMLV-miR21 tumors, as compared to pGMLV-ct tumors (*P* = 0.045 at Day 6 and *P* = 0.031 at Day 7, Fig. 6a). Consistent with in vitro study, p-STAT3-positive Treg cells, as well as ICOS/ICOSL-mediated interaction of Treg cells with endothelial cells, were increased in untreated pGMLV-miR21 tumors (Fig. 6b and c). Instead, Bcl-2-positive microvessels were reduced in pGMLV-miR21 tumors upon ABT-199 treatment (Fig. 6d), in parallel with the induction of apoptotic bodies in endothelial cells, as revealed by ultrastructural study (Fig. 6e).

## Discussion

MiR21 is a key regulator of disease progression in B-cell lymphoma [23, 24]. Experimentally, the overexpression of miR21 leads to a pre-B malignant lymphoid-like phenotype [25]. In clinical settings, increased circulating miR21 level in sera from DLBCL patients is associated with matched tumor tissue, advanced disease stage and inferior overall survival [26, 27]. More recently, gene ontology and pathway analysis has suggested that cell-environment interaction is another important miR21 pathogenic mechanism [28]. Here we not only confirmed miR21 as a serum oncogenic biomarker of DLBCL, but also provided a direct link of miR21 with lymphoma progression and tumor angiogenesis.

Dysregulated tumor microenvironment determines cancer cell chemosensitivity [29]. Our study showed that ectopic expression of miR21 led to chemoresistance of B-lymphoma cells, more profoundly when lymphoma cells were co-cultured with immune cells and endothelial cells, main components of tumor microenvironment. This is in accordance with previous studies which showed miR21 provokes myeloma cell adhesion to bone marrow stromal cells and resistance to chemotherapeutic agents [30], in addition, miR21 initiates inflammatory signaling in HER2-positive breast cancer and reduces the cytotoxic effect of neoadjuvant trastuzumab and chemotherapy [31].

Crosstalk between Treg cells and endothelial cells is essential in drug resistance [6]. ICOS, being a member of the CD28 family of co-stimulatory molecules, is implicated in maintaining durable immune reactions upon binding to ICOSL [32, 33]. Particularly, ICOS/ICOSL axis plays a pivotal role in Treg cell function and promotes Treg differentiation through activating the phosphoinositide 3-kinase-Akt pathway [34, 35]. MiR21 can modulate ICOS-ICOSL expression and contribute to the progression of colorectal cancer [36]. To our knowledge, we provided the first evidence that miR21 enhanced the interaction of Treg cells with endothelial cells, induced ICOS expression on Treg cells, stimulated tumor angiogenesis via ICOS/ICOSL signaling, and led to chemoresistance of B-cell lymphoma. Recent study showed that follicular lymphoma cells generate Treg cells via ICOS/ICOSL cascade and are susceptible to anti-ICOS/ICOSL therapy [37]. Therefore, our results highlighted a key role for Treg cells in DLBCL progression and suggested that targeting ICOS/ICOSL pathway may be an alternative immunotherapy for DLBCL treatment.

ABT-199 can specially target Bcl-2 on tumor cells and induces B-lymphoma cell apoptosis. However, it is unclear whether ABT-199 would affect tumor microenvironment in a Bcl-2-dependent manner. Here, both in vitro and in vivo, our study characterized a Bcl-2-dependent inhibition of ABT-199 on tumor angiogenesis, mediated by intrinsic miR21-Treg cell pathway, leading to sensitizing effect of ABT-199 on B-cell lymphoma. Therefore, in addition to the oncogenic role of miR21 on malignant B-lymphocytes [38], miR21 possessed a potential activity on tumor microenvironment, indicative an alternative mechanism of B-lymphoma cell response to ABT-199.

The above data demonstrated that miR21 plays an oncogenic role in B-cell lymphoma by modulating tumor microenvironment and supported clinical rationale for using miR21 as a biomarker to select chemoresistant B-lymphoma patients who may benefit from ABT-199 treatment.

## Additional files

Additional file 1: Figure S1. Cell apoptosis and cell cycle of SU-DHL-4 and SU-DHL-8 cells before and after ABT-199 treatment. A: ABT-199 induced cell apoptosis in SU-DHL-4, but not in SU-DHL-8 cells. B: No cell cycle arrest was observed in SU-DHL-4 and SU-DHL-8 upon ABT-199 treatment. (JPG 247 kb)

Additional file 2: Figure S2. ICOSL expression on HUVEC cells in the co-culture systems of SU-DHL-4 and SU-DHL-8 cells. ICOSL was stably expressed on HUVEC cells in the co-culture systems of SU-DHL-4 and SU-DHL-8 cells. (JPG 203 kb)

## Abbreviations

DLBCL: Diffuse large B-cell lymphoma
HUVEC: Human umbilical vein endothelial cell
ICOS: Inducible co-stimulator
ICOSL: ICOS ligand
IPI: International prognostic index
LDH: Lactate dehydrogenase
MiRs: MicroRNAs
Treg: Regulatory T cells
