# Supplementary figures and images for: MiR21 sensitized B-lymphoma cells to ABT-199 via ICOS/ICOSL-mediated interaction of Treg cells with endothelial cells
Source: J Exp Clin Cancer Res. 2017 Jun 21;36:82. doi: 10.1186/s13046-017-0551-z (PMC5480196; doi:10.1186/s13046-017-0551-z)

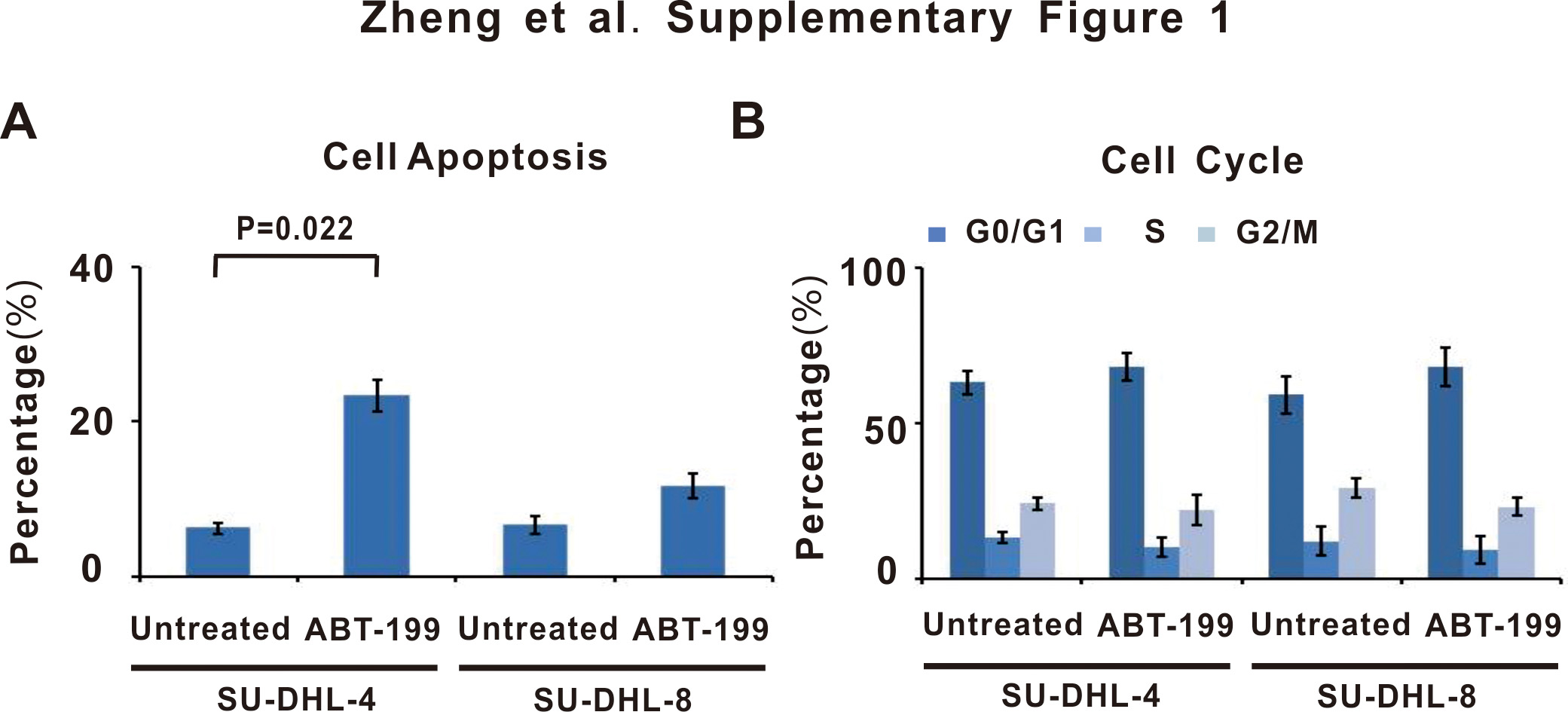

Supplement: Supplementary file 1 — Cell apoptosis and cell cycle of SU-DHL-4 and SU-DHL-8 cells before and after ABT-199 treatment. A: ABT-199 induced cell apoptosis in SU-DHL-4, but not in SU-DHL-8 cells. B: No cell cycle arrest was observed in SU-DHL-4 and SU-DHL-8 upon ABT-199 treatment. (JPG 247 kb) [file 13046_2017_551_MOESM1_ESM.jpg]

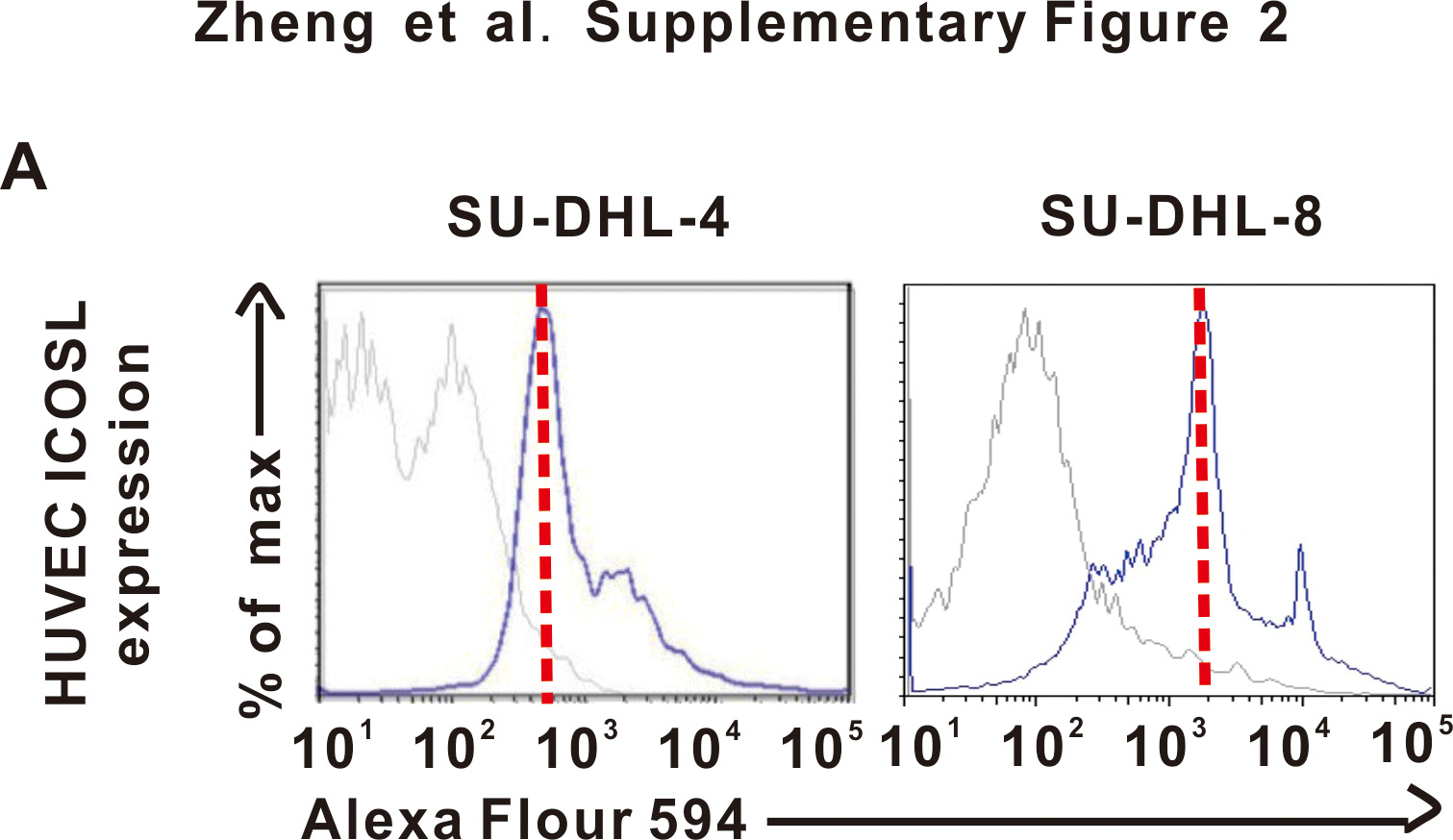

Supplement: Supplementary file 2 — ICOSL expression on HUVEC cells in the co-culture systems of SU-DHL-4 and SU-DHL-8 cells. ICOSL was stably expressed on HUVEC cells in the co-culture systems of SU-DHL-4 and SU-DHL-8 cells. (JPG 203 kb) [file 13046_2017_551_MOESM2_ESM.jpg]
